# Spike Protein Subunits of SARS-CoV-2 Alter Mitochondrial Metabolism in Human Pulmonary Microvascular Endothelial Cells: Involvement of Factor Xa

**DOI:** 10.1155/2022/1118195

**Published:** 2022-11-18

**Authors:** Khaoula Zekri-Nechar, José J. Zamorano-León, Carmen Reche, Manel Giner, Ana López-de-Andrés, Rodrigo Jiménez-García, Antonio J. López-Farré, Carlos Hugo Martínez-Martínez

**Affiliations:** ^1^School of Medicine, Universidad Complutense, Madrid, Spain; ^2^Public Health and Maternal, Child Health Department, School of Medicine, Universidad Complutense, Madrid, Spain; ^3^IdISSC, Madrid, Spain; ^4^Gomez Ulla Central Defense Hospital, Madrid, Spain; ^5^Surgical Departments, School of Medicine, Universidad Complutense, Madrid, Spain

## Abstract

**Background:**

Mitochondria have been involved in host defense upon viral infections. Factor Xa (FXa), a coagulating factor, may also have influence on mitochondrial functionalities. The aim was to analyze if in human pulmonary microvascular endothelial cells (HPMEC), the SARS-CoV-2 (COVID-19) spike protein subunits, S1 and S2 (S1+S2), could alter mitochondrial metabolism and what is the role of FXA.

**Methods:**

HPMEC were incubated with and without recombinants S1+S2 (10 nmol/L each).

**Results:**

In control conditions, S1+S2 failed to modify FXa expression. However, in LPS (1 *μ*g/mL)-incubated HPMEC, S1+S2 significantly increased FXa production. LPS tended to reduce mitochondrial membrane potential with respect to control, but in higher and significant degree, it was reduced when S1+S2 were present. LPS did not significantly modify cytochrome c oxidase activity as compared with control. Addition of S1+S2 spike subunits to LPS-incubated HPMEC significantly increased cytochrome c oxidase activity with respect to control. Lactate dehydrogenase activity was also increased by S1+S2 with respect to control and LPS alone. Protein expression level of uncoupled protein-2 (UCP-2) was markedly expressed when S1+S2 were added together to LPS. Rivaroxaban (50 nmol/L), a specific FXa inhibitor, significantly reduced all the above-mentioned alterations induced by S1+S2 including UCP-2 expression.

**Conclusions:**

In HPMEC undergoing to preinflammatory condition, COVID-19 S1+S2 spike subunits promoted alterations in mitochondria metabolism suggesting a shift from aerobic towards anaerobic metabolism that was accompanied of high FXa production. Rivaroxaban prevented all the mitochondrial metabolic changes mediated by the present COVID-19 S1 and S2 spike subunits suggesting the involvement of endogenous FXa.

## 1. Introduction

The severe acute respiratory syndrome coronavirus type 2, SARS-CoV-2 (COVID-19), emerged in 2019. COVID-19 causes a range of respiratory symptoms but also stimulates coagulability as the severity of COVID-19 increases. In fact, it is widely established that COVID-19 infection courses with a thrombo-coagulant state, favoring venous thromboembolic risk and inflammatory storm, which may result in pneumonia, acute respiratory distress syndrome, and sepsis [1, 2].

The critical role of coagulation pathways in the pathogenesis of severe acute respiratory syndrome coronavirus SARS-CoV-2 (COVID-19) has been widely supported by large number of studies, being described as close relationship between alterations of several biomarkers of coagulation activation and disease severity, including factor Xa (FXa) [3, 4]. FXa is a serine protease coagulating factor expressed in several cells including platelets, alveolar cells, bronchiolar epithelium, and cells of vascular vessels [5, 6]. Interestingly, the endogenous overexpression of FXa during serious SARS-CoV-2 infection seems to contribute to the pathogenesis and complications of COVID-19 [7, 8].

To enter human host cells, COVID-19 uses its spike glycoprotein. The spike protein consists of two subunits: subunit 1 (S1), containing host angiotensin-converting enzyme 2 (ACE2) receptor-binding domain, and the subunit 2 (S2) that plays important role in the fusion process of COVID-19 to host cells [9]. In addition to coagulant effects of FXa, it has been suggested that protease activity of FXa may also be important for viral fusion and entry of SARS-CoV [10, 11]. Indeed, it has been described that FXa inhibition blocked the viral entry of SARS-CoV into the host cells by preventing the spike protein cleavage into the S1 and S2 subunits [12]. Direct FXa inhibitors may lead to be considered as a potential promise in treating COVID-19 because of their anticoagulant, anti-inflammatory, and antiviral activities [13].

Pathological situations such as pulmonary diseases, diabetes mellitus, and obesity are closely associated with mitochondrial functionality alterations [14, 15]. Several pieces of evidence have suggested that mitochondria also function as platform for host response upon viral infections and immunity response [16]. As example, reported data have demonstrated that influenza A, herpes, and hepatitis B and C viruses, among others, promote changes in mitochondrial dynamic for persistent infection [17, 18]. It has been suggested that SARS-CoV-2 has the ability to compromise mitochondrial function in peripheral blood mononuclear cells, contributing to systemic immune response [19]. Recently, an in vitro study described that treatment with both SARS-COV 2 spike protein induced mitochondrial dysfunction in microglial cells [20]. Interestingly, further to participate in coagulation, FXa acts on the mitochondrial functionality changing the expression and activities of mitochondrial proteins, including proteins related to mitochondrial energetic metabolism. In this regard, in human abdominal aortic aneurysms, our group recently demonstrated that rivaroxaban, an oral specific inhibitor of FXa, improved mitochondrial functionality which was associated with changes in mitochondrial proteins related to mitophagy [21].

Taken together, the aim of the present work was to analyze if in human pulmonary microvascular endothelial cells, the COVID-19 spike protein subunits, S1 and S2, may alter mitochondrial functionality and if endogenous FXa could be involved.

## 2. Material and Methods

### 2.1. Human Pulmonary Microvascular Endothelial Cell Culture

Human pulmonary microvascular endothelial cells (HPMEC) were purchased from ScienCell Research Laboratories (Carlsbad, CA, USA).

Cells were cultured in accordance with the manufacturers' instructions in 5% CO_2_ at 37°C. Cells were used at passages 3–6, and they were maintained in low fetal bovine serum (0.5%)-containing medium overnight before the experiment.

Cells were incubated with a cocktail containing the recombinant COVID-19 spike S1 subunit protein, which contain both the entire S1 (Val16-Gln690) and the S2 subunits (Met697-Pro1213). The final concentration of each COVID-19 spike subunit was 10 nmol/L, and it was based on previous reports from Suzuki et al. showing that a similar COVID-19 spike concentration activates the MEK/ERK pathway and Buzhdygan et al. reporting in an in vitro model that this S1 and S2 concentration alters blood-brain barrier [22, 23].

To simulate a preinflammatory situation, experiments were also performed with Escherichia coli lipopolysaccharide (LPS 1 *μ*g/mL, catalog#L2630, Sigma-Aldrich, St. Louis, MO, USA).

The role of endogenous FXa was analyzed by incubating HPMEC with 50 nmol/L rivaroxaban (Bay 59–7939, diluted in dimethyl sulfoxide, 1% final concentration). Equal amount of dimethyl sulfoxide (1%) was also added to the other experimental groups. This rivaroxaban concentration was chosen based on a previous works showing that 50 nmol/L rivaroxaban inhibited thrombin formation [9]. It is remarkable that 50 nmol/L rivaroxaban is equivalent to approximately 200 *μ*g/L rivaroxaban concentration that is reached in patients treated with daily dose of 20 mg rivaroxaban [24].

All incubations with the HPMEC were carried out under sterile conditions.

### 2.2. Western Blot Analysis

As previously reported [25], HPMEC was homogenized in a lysis buffer containing 8 mol/L urea, 2% CHAPS *w*/*v*, and 40 mmol/L dithiothreitol. Proteins from HPMEC homogenates were then separated by electrophoresis loading equal amounts of total protein (20 *μ*g/lane), measured by the bicinchoninic acid kit (Pierce Rockford, IL, USA), and running onto denaturing SDS-PAGE 12% (*w*/*v*) polyacrylamide gels.

Thereafter, gels were blotted onto nitrocellulose membranes and were incubated with 5% (*w*/*v*) bovine serum albumin. Nitrocellulose membranes were then incubated with a polyclonal antibody against FXa (12255–05021, dilution 1 : 800; AssayPro, St. Charles, MO, USA) and uncoupled protein-2 (UCP-2, 1 : 1000; PA5-36383; Invitrogen; Thermo Fisher Scientific, Waltham, MA, USA). Nitrocellulose membranes were also incubated with a monoclonal antibody against lactate dehydrogenase (LDH, 1 : 1000, Sc-1333123 Santa Cruz Biotechnology, Inc.).

To verify that equal amounts of proteins were loaded in the gel, parallel gel with identical samples was run and the expression of the constitutive protein *β*-actin was detected as loading control (*β*-actin antibody 1 : 1500, A-5441 Sigma-Aldrich, St. Louis).

Nitrocellulose membranes were revealed with peroxidase-conjugated anti-rabbit IgG (1 : 2000) for UCP-2 and FXa (1 : 2500) and peroxidase-conjugated anti-mouse IgG for LDH and *β*-actin (1 : 2500).

The chemiluminescence signal was obtained using chemiluminescence reagents (ECL; GE Healthcare, Little Chalfont Buckinghamshire, UK), and it was detected and analyzed using the iBright Imaging System (iBright FL100, Thermo Fisher Scientific, MA, USA).

### 2.3. Citrate Synthase, Cytochrome C Oxidase, and Lactate Dehydrogenase Activities

Measurements of citrate synthase, cytochrome c oxidase, and LDH activities were done using colorimetric commercial kits following the manufacturer's instructions.

Citrate synthase activity was determined using a colorimetric commercial kit (CS0720, Sigma-Aldrich, St. Louis, MO, USA) based on the conversion of acetyl-CoA and oxaloacetic acid into citric acid. The hydrolysis of the thioester of acetyl CoA results in the formation of CoA with a thiol group that forming 5-thio-2-nitrobenzoic acid with 5,5-dithiobis-(2-nitrobenzoic acid) is spectrophotometrically detected at 412 nm. An amount of 8 *μ*g of total protein/sample was used in the experiment.

Cytochrome c oxidase activity was determined by a colorimetric commercial kit (109911, Abcam, Cambridge, UK) based on the oxidation of reduced cytochrome c as an absorbance decrease at 550 nm. An amount of 20 *μ*g of total protein/sample was used in the experiment.

LDH activity was also determined with a colorimetric commercial kit (K726-50, BioVision, Milpitas, CA. USA) based on reduction of NAD to NADH, which then interacts with a probe to produce a detected color at 450 nm. An amount of 25 *μ*g of total protein/sample was used in the experiment.

### 2.4. Changes in Mitochondrial Membrane Potential (ΔΨ*m*)

Changes in ΔΨ*m* were analyzed using a JC-10 fluorometric assay kit (MAK159 Sigma-Aldrich, St. Louis, MO, USA) according to the manufacturer's protocol. In brief, JC-10 dye loading solution (final concentration 2 *μ*g/mL) was added to HPMEC that were incubated in the dark for 45 min, at 37°C. Fluorescence signal was detected using the iBright Imaging System. The ratio of fluorescence intensities at 590 nm/525 nm was used to determine mitochondrial membrane depolarization.

### 2.5. Mitochondria Isolation and Mitochondrial S1 and S2 Detection

Mitochondrial extracts from HPMEC were obtained using a commercial mitochondria isolation kit (catalog#89874, Thermo Fisher) following the manufacturer's specifications. Isolated mitochondria from HPMEC were lysates in the above-mentioned lysis buffer and centrifuged at 12,000*g* for 10 min.

To detect the possible mitochondrial content of S1 and S2 spike subunit COVID-19 proteins, Western blot and dot blot techniques were used. Western blots were performed as mentioned above, and for the dot blots, mitochondrial homogenates were spotted (5 *μ*g/spot/sample) onto nitrocellulose membranes, as positive control recombinant spike subunits S1 and S2 were also loaded.

Nitrocellulose membrane was blocked with 5% (*w*/*v*) bovine serum albumin and then incubated with a polyclonal antibody against S1 (1 : 1000, PA5-81795, Invitrogen, Thermo Fisher) and a monoclonal antibody against S2 (1 : 1000, MAB10557, R&D System) spike subunit proteins. Membranes were then incubated with peroxidase-conjugated anti-rabbit IgG for S1 subunit antibody and peroxidase-conjugated anti-mouse antibody for S2 subunit and developed using chemiluminescence reagents (ECL; GE Healthcare, Little Chalfont Buckinghamshire, UK). Protein expression level was detected by an iBright Imaging System (iBright FL100, Thermo Fisher Scientific).

### 2.6. Statistical Analysis

Values are expressed as mean ± standard error of mean (S.E.M). The expression levels of the different analyzed proteins and the enzymatic activities were compared by the nonparametric Mann–Whitney test. For the statistical analysis, the SPSS 25.0 software was used and a *p* value < 0.05 was considered as statistically significant.

## 3. Results

### 3.1. Changes in the Expression of FXa and Oxidative Stress-Related Proteins

**Figure 1:**
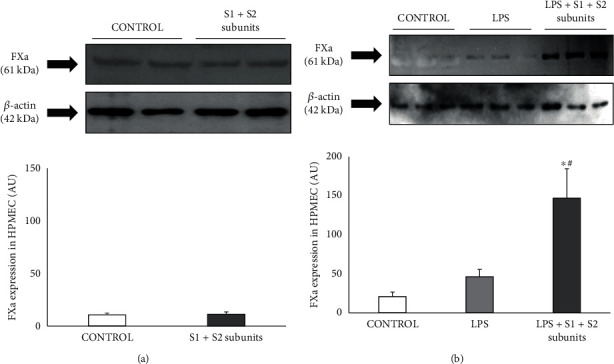
(a) Representative Western blot of the factor Xa (FXA) expression in human pulmonary microvascular endothelial cells (HPMEC) incubated in the absence (control) and in the presence of recombinant COVID-19 spike S1 and S2 subunits (10 nmol/L, each subunit; S1+S2 subunits). (b) Representative Western blot of FXA expression in HPMEC under control condition and incubated with LPS (1 *μ*g/mL) with and without recombinant COVID-19 spike S1 and S2 subunits (10 nmol/L, each subunit) (LPS+S1+S2 subunits). Bar graphs show the densitometric analysis of all the Western blots represented as densitometric arbitrary units (AU). Densitometric values are represented as mean ± SEM of four experiments. ^∗^*p* < 0.05 respect to control. ^#^*p* < 0.05 respect to LPS.

In control HPMEC, the presence of COVID-19 spike subunits S1+S2 did not modify the expression level of FXa protein with respect to control (Figure 1(a)).

Experiments were then performed under a preinflammatory condition. For this purpose, HPMEC were incubated with LPS (1 *μ*g/mL). As Figure 1(b) shows, the incubation of HPMEC with 1 *μ*g/mL LPS did not significantly modify the content of FXa in HPMEC with respect to control. However, COVID-19 spike subunits S1+S2 markedly increased FXa protein expression in LPS-incubated HPMEC as compared with either control or LPS alone (Figure 1(b)).

### 3.2. Changes in ΔΨ*m*

**Figure 2:**
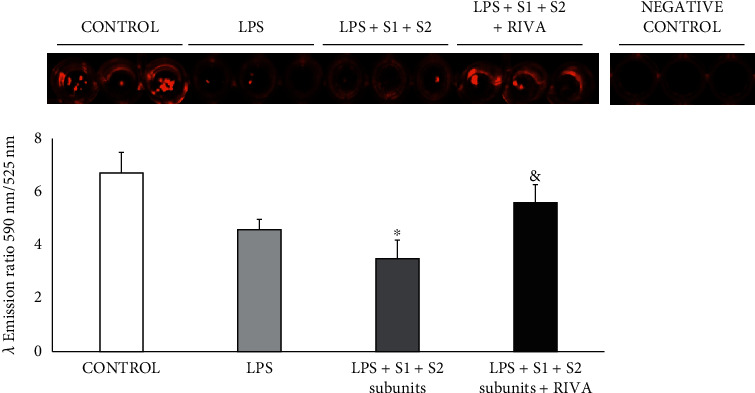
Representative cell fluorescent changes in mitochondrial membrane potential (ΔΨ*m*). Results are represented as mean ± SEM of four experiments. ^∗^*p* < 0.05 respect to control. ^&^*p* < 0.05 respect to LPS+S1+S2 subunits.

Incubation of HPMEC with LPS nonsignificantly reduced the ratio 590 nm/525 nm with respect to control (Figure 2). Addition of COVID-19 spike subunits S1+S2 reduced in higher degree the ratio 590 nm/525 nm of ΔΨ*m* than that observed with LPS alone, which reached statistical significance with respect to control (Figure 2). In the presence of LPS and the two spike subunits, addition of 50 nmol/L rivaroxaban reverted the reduction of ΔΨ*m* observed without rivaroxaban, being reached similar ΔΨ*m* values to control (Figure 2).

### 3.3. Citrate Synthase, Cytochrome c Oxidase, and Lactate Dehydrogenase Activities

**Figure 3:**
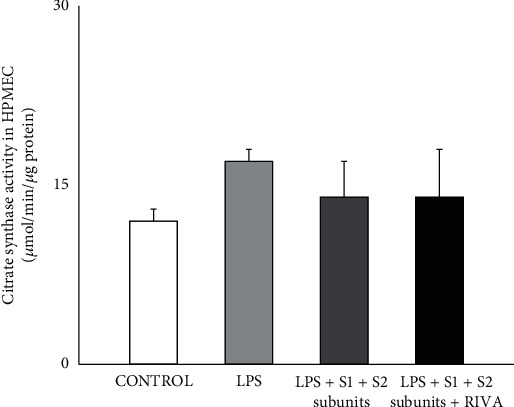
Citrate synthase activity in human pulmonary microvascular endothelial cells (HPMEC) incubated under control condition and in presence of LPS (1 *μ*g/mL) with and without recombinant COVID-19 spike S1 and S2 subunits (10 nmol/L, each subunit) (LPS+S1+S2 subunits). The effect of rivaroxaban (50 nmol/L) on LPS-incubated HPMEC with COVID-19 spike S1 and S2 subunits was also tested. Results are represented as mean ± SEM of four experiments.

Citrate synthase activity was similar among control, LPS-, and LPS+COVID-19 spike S1 + S2 subunit-incubated HPMEC (Figure 3). Moreover, in LPS-incubated HPMEC and with the presence of recombinant COVID-19 S1+S2 spike subunits, rivaroxaban did not modify citrate synthase activity with respect to the other experimental groups (Figure 3).

**Figure 4:**
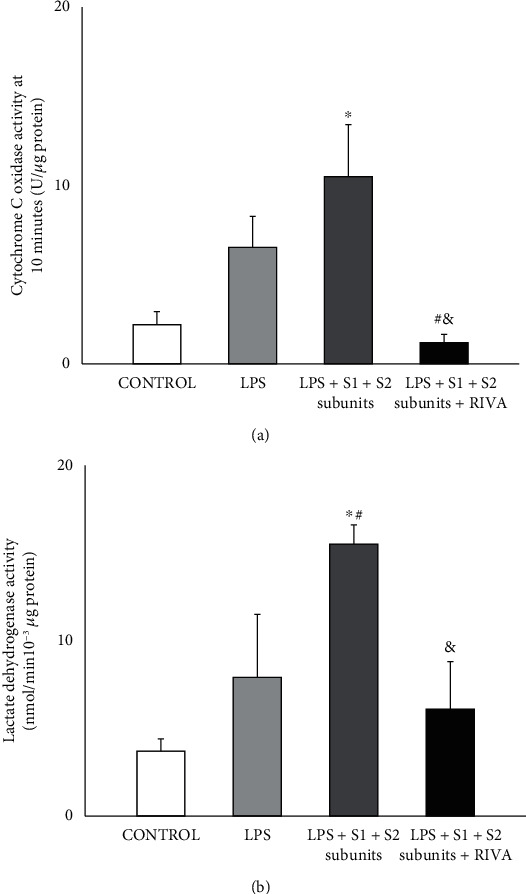
Cytochrome C oxidase (a) and lactate dehydrogenase (b) activities in HPMEC incubated under control condition and in the presence of LPS (1 *μ*g/mL) with and without recombinant COVID-19 spike S1 and S2 subunits (10 nmol/L, each subunit) (LPS+S1+S2 subunits). The effect of rivaroxaban (50 nmol/L) on LPS-incubated HPMEC with COVID-19 spike S1 and S2 subunits was also examined. Results are represented as mean ± SEM of four experiments. ^∗^*p* < 0.05 respect to control. ^#^With respect to LPS alone. ^&^*p* < 0.05 respect to LPS+S1+S2 subunits.

In HPMEC, cytochrome c oxidase activity tended to be increased by LPS although it did not reach statistical significance with respect to control (Figure 4(a)). However, addition of COVID-19 S1+S2 spike subunits to LPS-incubated HPMEC increased cytochrome c oxidase activity reaching statistical significance with respect to control (Figure 4(a)).

In HPMEC incubated with LPS+COVID-19 S1+S2 spike subunits, rivaroxaban completely reduced cytochrome c oxidase activity to similar levels than that found in control and statistically lower than in those observed in HPMEC incubated with either LPS alone or with LPS+COVID-19 S1+S2 spike subunits (Figure 4(a)).

In HPMEC, LDH activity also tended to be increased by LPS although, as compared with control, it did not reach statistical significance (Figure 4(b)). However, a significant increase in LDH activity was found after addition of both COVID-19 spike S1+S2 subunits and LPS. This increased LDH activity was statistically significant with respect to either control or LPS alone (Figure 4(b)).

In the HPMEC coincubated with COVID-19 S1+S2 spike subunits and LPS, the presence of rivaroxaban significantly reduced the observed increment of LDH reaching similar levels than that in control (Figure 4(b)).

**Figure 5:**
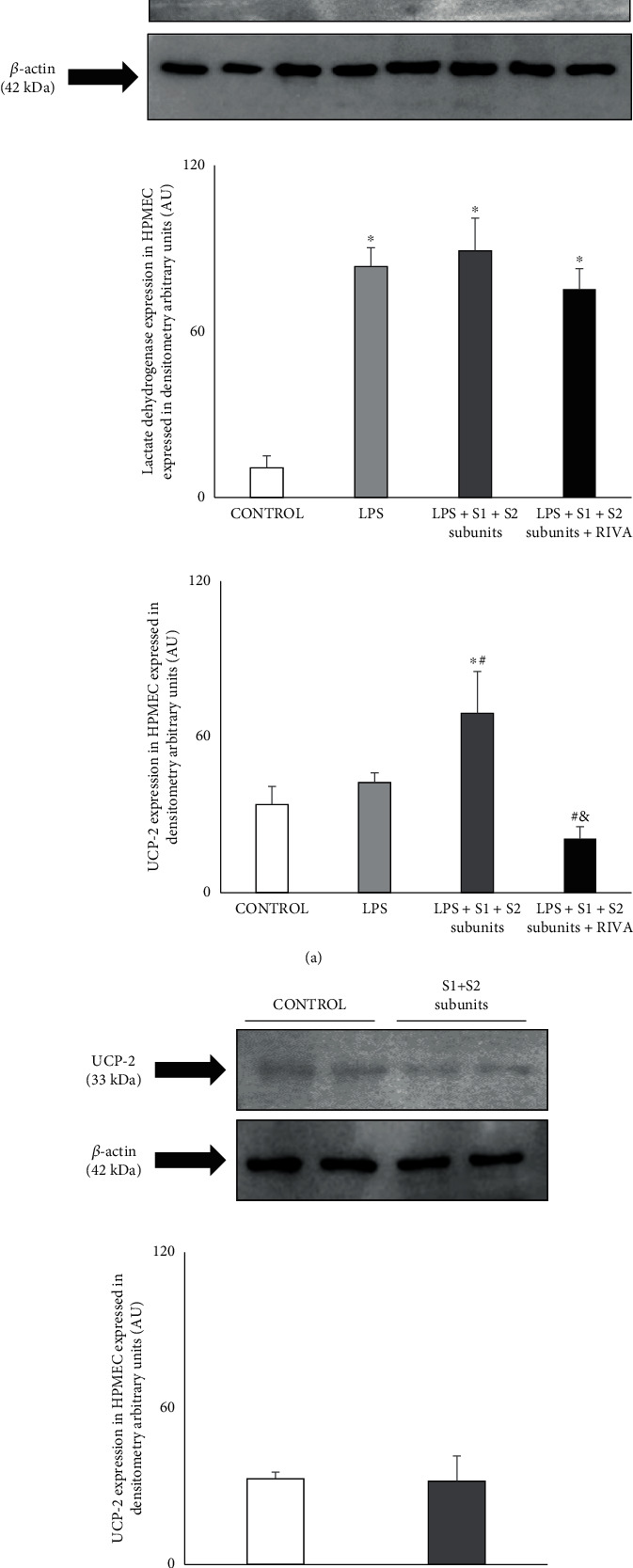
(a) Representative Western blot showing lactate dehydrogenase (LDH) and UCP-2 expression in HPMEC incubated under control condition and in the presence of LPS (1 *μ*g/mL) with and without recombinant COVID-19 spike S1 and S2 subunits (10 nmol/L, each subunit) (LPS+S1+S2 subunits). The effect of rivaroxaban (50 nmol/L) on LPS-incubated HPMEC with COVID-19 S1 and S2 spike subunits is also shown. (b) Representative Western blot of UCP-2 expression in HPMEC incubated without LPS but with the presence (S1+S2 subunits) and the absence of the recombinant COVID-19 spike S1 and S2 subunit proteins (10 nmol/L, each subunit). At the bottom, the densitometric analysis showed as arbitrary densitometric units (AU) of the corresponding Western blot. Results are represented as mean ± SEM of three experiments. ^∗^*p* < 0.05 respect to control. ^#^*p* < 0.05 respect to LPS. ^&^*p* < 0.05 respect to LPS+S1+S2 spike subunits.

In HPMEC, LPS significantly increased LDH expression (Figure 5(a)). In LPS-incubated HPMEC, similar increase of LDH expression was found in the presence of either the COVID-19 spike subunits with and without rivaroxaban (Figure 5(a)).

In both control condition and LPS-incubated HPMEC, UCP-2 expression was almost undetectable (Figure 5(a)). However, a significant increase in UCP-2 protein expression was observed when COVID-19 spike S1 and S2 subunits were added to LPS-incubated HPMEC (Figure 5(a)). Addition of rivaroxaban to LPS+S1+S2 spike subunit-incubated HPMEC markedly reduced UCP-2 content and turned him back to almost undetectable (Figure 5(a)).

In the HPMEC and in the absence of LPS, COVID-19 S1+S2 spike subunits by themselves failed to modify UCP-2 expression (Figure 5(b)).

### 3.4. Absence of S1 and S2 Spike Subunit Interaction with Mitochondria

It was analyzed if, in HPMEC, S1 and/or S2 COVID-19 spike subunits could be bound to mitochondria. In Western blot analysis, neither S1 nor S2 was detected in the mitochondria isolated from LPS-stimulated HPMEC and incubated with S1 and S2 subunits proteins (data not shown).

**Figure 6:**
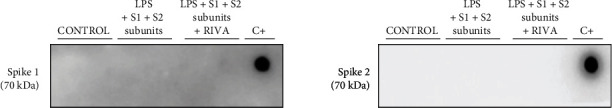
Representative dot blots to identify S1 and S2 spike subunits interacting with mitochondria from HPMEC. Experiments were performed in HPMEC incubated under control condition and in the presence of LPS (1 *μ*g/mL) with recombinant COVID-19 spike S1 and S2 subunits (10 nmol/L, each subunit) (LPS+S1+S2 subunits). The effect of rivaroxaban (50 nmol/L) was also analyzed. Recombinant COVID-19 spike S1 or S2 subunits were, respectively, loaded as positive control (C^+^).

It could be then occurring that due to a possible relative low level of S1 and S2 subunits in the mitochondria, they could not be detectable by the Western blotting technique. Therefore, experiments using dot blot were then performed. However, dot blot experiments also failed to detect either S1 or S2 spike subunits in isolated mitochondria from LPS-stimulated HPMEC incubated with S1 and S2 subunit proteins (Figure 6).

## 4. Discussion

The present work shows that COVID-19 spike S1+S2 subunits had ability to alter cytochrome c oxidase and LDH activities, mitochondrial membrane potential, and the level of expression of mitochondrial UCP-2 protein in HPMEC undergoing to a preinflammatory condition, simulated by addition of LPS. The FXa expression in HPMEC was significantly increased after addition of COVID-19 spike subunits S1 and S2 and LPS. Moreover, all these mitochondrial effects associated with the presence of COVID-19 spike subunits were inhibited by the FXa inhibitor rivaroxaban, suggesting the involvement of endogenous FXa in the mitochondrial effects elicited by the COVID-19 spike subunits.

The COVID-19 spike protein consists of two subunits: subunit 1 (S1) that contains the ACE2 receptor-binding domain and subunit 2 (S2) involved in the fusion process of the spike protein with ACE2 receptors [8]. Therefore, it is generally thought that the sole function of the COVID-19 spike protein is to allow COVID-19 virus binding to host cells. Interestingly, a previous work using the recombinant SARS-CoV-1 spike protein, that it is 76-78% identical to the SARS-CoV-2 spike protein [26], increased in mice lung angiotensin II production and inhibition of angiotensin II type 1 receptors attenuated the enhancement of lung injury associated with the SARS-CoV-1 spike protein [27]. Therefore, it would be plausible to think that SARS-CoV2 spike proteins had ability to exert effects on host cells additional to binding function. In this regard, analyzing the activity of enzymes-related to ATP production and as compared to HPMEC incubated with LPS alone, COVID-19 spike S1+S2 subunits promoted on LPS-incubated HPMEC the following changes: 1.- Reduction of *ΔΨ*m; 2.- Enhancement of cytochrome c oxidase activity; 3.- Enhancement of LDH activity without changes in LDH expression; 4.-Increased expression of mitochondrial UCP-2 protein. However, it is important to point out that without preinflammatory condition, COVID-19 spike subunits by themselves failed to modify UCP-2 expression in HPMEC suggesting that only under preinflammatory conditions, COVID-19 spike subunit protein may modify mitochondrial activities.

Cytochrome c oxidase (complex IV) is the last electron acceptor of the respiratory chain and major oxygen consumer enzyme in the cell, representing the rate-limiting step of the mitochondrial electron transport chain [28]. In our experiments, cytochrome c oxidase activity was enhanced by COVID-19 spike S1+S2 subunits when they were together added to LPS to HPMEC. Citrate synthase activity was not modified by incubating HPMEC with LPS alone or with LPS+S1+S2 subunits. Citrate synthase activity enzyme, used as marker of mitochondria density, was not different among the control, LPS, and LPS + S1 + S2 groups [29]. Therefore, the finding of the increased cytochrome c oxidase activity should be not explained by changes in mitochondrial density. As speculation, the increased cytochrome c oxidase activity could be reflecting an attempt of cytochrome c oxidase to favor mitochondrial ATP formation since UCP-2 expression was also increased. In this regard, there are two main mechanisms regulating cytochrome c oxidase activity. The first of these is the reduction of ΔΨ*m*. Substrate-derived electrons from glucose and fatty acid metabolism flow through complexes I to IV of the electron transport chain, and released energy is used for pumping protons (H^+^) from the matrix into the intermembrane space. The resulting proton gradient sustains the ΔΨ*m*, which drives ATP synthase. Reduction of ΔΨ*m* stimulates mitochondrial respiratory chain [30]. In the experiments, COVID-19 spike S1+S2 subunits reduced ΔΨ*m* even more than that produced with LPS alone. The second control mechanism, closely related to ΔΨ*m*, is ATP by itself. The high-affinity binding of ATP to the matrix domain of complex IV induces an allosteric ATP inhibition of cytochrome c oxidase activity. In this regard, in LPS-incubated HPMEC, the protein expression level of UCP-2 protein was markedly enhanced by COVID-19 spike S1+S2 subunits. It is well established that UCP-2 protein induces proton leak, resulting lower values ofΔΨ*m*and diminished ATP production associated with heat generation. Therefore, it could be plausible that lesser ATP levels could be forming due to proton leaking related to UCP-2, which may favor glycolysis and, therefore, the stimulation of LDH activity. Accordingly, LDH activity was higher in LPS-incubated HPMEC in the presence of the COVID-19 spike subunits.

All these results suggest that under preinflammatory condition, COVID-19 spike S1+S2 subunits promote by themselves the alterations in the mitochondrial energetic metabolism shifting from aerobic condition towards anaerobic metabolism. Interestingly, anaerobic respiration favors pyruvate reduction into lactate that is ensured by lactate dehydrogenase (LDH), which seems to be highly upregulated marker in COVID-19 illness [31]. A recent analysis revealed the downregulation of genes involved in mitochondrial aerobic metabolism genes in the SARS-CoV-2 infected lung cell lines, suggesting that mitochondrial disruption and anaerobic metabolism increased inflammation and severity in the COVID-19-related sepsis [32].

Different works have reported a significant increase in the amount of FX, FXa precursor in COVID-19 patients [33, 34]. FXa plays an important role in coagulation, but FXa is not only involved in coagulation but also as a stimulator of inflammation and oxidative stress, both mechanisms associated with the worse outcome of COVID-19-infected patients [35, 36]. In addition, our group recently reported mitochondrial effects of FXa in human abdominal aortic aneurysmal site, most of them prevented by rivaroxaban, a specific FXa inhibitor [21].

In the present study, it was found that COVID-19 spike S1+S2 subunits markedly increased the expression of FXa protein in LPS-stimulated HPMEC. Therefore, it was analyzed if FXa may be involved in the mitochondrial effects observed in the HPMEC incubated with LPS and COVID-19 spike S1+S2 subunits. Interestingly, our results revealed that the presence of rivaroxaban had ability to revert the mitochondrial effects induced by COVID-19 spike S1+S2 subunits, reducing both cytochrome c oxidase and LDH activities. It was also accompanied of greater reduction of ΔΨ*m*. Moreover, rivaroxaban also prevented the increase in UCP-2 expression observed in HPMEC incubated with COVID-19 spike S1+S2 subunits and LPS. These changes could not be attributed to modifications of mitochondrial density since citrate synthase activity was similar to that found in the other experimental groups. Taken all together, these results suggest the involvement of endogenous FXa in the deleterious effects on the mitochondrial energetic metabolism promoted by the COVID-19 spike S1+S2 subunits in HPMEC submitted to preinflammation.

Molecular pathway by which COVID-19 spike S1+S2 subunits would be able to affect mitochondria functionality remains unknown. In a machine-learning model study, Wu et al. suggested that the 5′ and 3′ untranslated regions of COVID-19 could be localized into the mitochondria although this hypothesis has not been experimentally evaluated [37]. It also suggested the possibility that the SARS-CoV-1 nonstructural protein may interact with mitochondrial DNA- (mtDNA-) encoded complex IV, opening the possibility that some virus proteins may directly interact with mitochondria [38]. However, neither Western blot nor dot blot experiments detected either S1 or S2 spike subunits in isolated mitochondria from LPS+S1+S2 spike subunit-incubated HPMEC. Although evidently it should be not discarded at all the possibility that the COVID-19 spike subunits of the spike protein may interact with the mitochondria, however, these observations diminished such possibility.

### 4.1. Comments and Study Limitations

We are aware that there are many unresolved questions raised from this study. Probably, the first limitation is that the present experimental design does not allow us to know whether independently S1, S2, or both spike subunits are needed to induce alterations in the mitochondria. Future studies are then warranted. Although it is beyond the scope of the present work, it is also important to point out that FXa is a serine protease [39]. As mentioned, it is known that the COVID-19 spike protein needs to be cleaved into its subunits by host enzymes for viral entry into host cells. Therefore, it could be plausible that FXa could also cleave COVID-19 spike S1 and S2 subunits favoring viral host infection as it was reported for another coronavirus [12]. Even more, a recent review also speculated that FXa could cleavage the COVID-19 spike protein into its subunits increasing the COVID-19 infectively; however, at present, there were no experimental studies about it. However, our experimental design discards that the obtained results were due to modifications in COVID-19 infectively by FXa activity since the experiments were performed with a cocktail containing independent recombinant S1 and S2 spike subunits.

On the other hand, in the present study, LPS was used with the only purpose to simulate a preinflammatory condition thus enabling us to analyze if COVID spike subunits may modify mitochondrial energy metabolism under this preinflammatory situation linked to COVID illness. In no case was the study intended to analyze the mitochondrial effects of LPS.

The importance of this work lies in that COVID-19 spike S1+S2 subunits themselves seem to have the ability to decrease mitochondrial functionality. It suggests the need to further explore the possible impact that COVID-19 spike subunits may have in population with previously compromised mitochondrial function such as in diabetes mellitus, sedentary lifestyle, or elderly patients suffering from an additional inflammatory condition. In addition, although there is no doubt about the importance of vaccination to stop COVID-19 pandemic, it should be kept in mind that individuals vaccinated with RNA and viral-vector-based vaccines use human cells to produce spike protein. Then, it should be very important to know more in depth the cellular effects of the COVID-19 spike subunits to prevent possible long-term consequences on health. Moreover, although in our knowledge FXa inhibitors as rivaroxaban have not been used as therapeutic alternative to prevent coagulopathies induced by COVID-19, it probably has a place for the treatment of hospitalized COVID-19 patients with low bleeding risk, particularly considering other reported properties of rivaroxaban such as its anti-inflammatory, antioxidant properties, and now probably its ability to protect mitochondria of HPMEC from the COVID-19 spike subunits.

As conclusion, in LPS-incubated HPMEC, COVID-19 spike S1 and S2 subunits promoted greater reduction of ΔΨ*m* and increase of cytochrome c oxidase and LDH activities accompanied of increase of UCP-2 expression suggesting a possible energetic metabolism change from aerobic towards anaerobic situation. Under preinflammatory condition, COVID-19 spike S1 and S2 subunits increased the expression level of FXa, and rivaroxaban, the FXa inhibitor, prevented the above-mentioned mitochondrial effects elicited by the presence of the COVID-19 spike subunits supporting the involvement of endogenous FXa. The present study is merely descriptive; future experiments are warranted to explore mechanistic pathways of potential COVID-19 spike S1 and S2 subunit effects on mitochondria and the role of FXa.

## Data Availability

Data to support the findings of this study is available on reasonable request from the corresponding author.
